# Herpes Simplex Virus Type 1 Encephalitis in a Six-Year-Old Girl: A Case Report From Latin America

**DOI:** 10.7759/cureus.95229

**Published:** 2025-10-23

**Authors:** Jorge Martinez-Vasquez, Alberto Vargas-Solano, Jeaustin Mora-Jiménez, Kevin Cruz-Mora, Esteban Zavaleta-Monestel

**Affiliations:** 1 Pediatrics and Child Health, Hospital Clinica Biblica, San José, CRI; 2 Diagnostic Radiology, Hospital Clinica Biblica, San José, CRI; 3 Department of Research, Hospital Clinica Biblica, San José, CRI; 4 Pharmacy, Hospital Clinica Biblica, San José, CRI

**Keywords:** acyclovir therapy, cerebrospinal fluid analysis, electroencephalography findings, herpes simplex virus encephalitis, magnetic resonance imaging, pediatric viral encephalitis

## Abstract

Herpes simplex virus type 1 (HSV-1) encephalitis is a severe neurological condition that can cause significant morbidity and mortality, particularly in pediatric patients. Prompt recognition and treatment are crucial to preventing long-term complications. This case report aims to highlight the diagnostic challenges and importance of early antiviral therapy in pediatric HSV-1 encephalitis. We describe a six-year-old girl admitted with fever, headache, vomiting, progressive somnolence, and a prolonged generalized tonic-clonic seizure. Upon admission, she exhibited oral aphthae, somnolence, and mild hyperkalemia. Brain magnetic resonance imaging revealed unilateral hyperintensity in the right mesial temporal lobe involving the hippocampus and parahippocampal gyrus. Electroencephalography demonstrated diffuse cortical dysfunction without epileptiform discharges. Cerebrospinal fluid polymerase chain reaction confirmed HSV-1 infection, while other inflammatory parameters remained within normal limits. Empirical intravenous acyclovir was initiated immediately and continued for 21 days, with favorable tolerance and neurological improvement. At the time of last follow-up, the patient remained clinically stable, with plans for long-term neurological monitoring. This case underscores the importance of early diagnosis and comprehensive management, including neuroimaging, electroencephalography, and cerebrospinal fluid analysis, to guide timely therapeutic decisions and prevent irreversible neurological damage in pediatric patients.

## Introduction

Encephalitis is an inflammation of the brain parenchyma that leads to a variety of neurological manifestations. This condition can result from multiple causes, both infectious and non-infectious. Among the most clinically relevant infectious agents worldwide is herpes simplex virus type 1 (HSV-1), recognized as one of the leading causes of acute sporadic viral encephalitis. This form of the disease stands out as the most common and potentially fatal variant [1,2].

In the United States, it is estimated that approximately seven individuals per 100,000 population are hospitalized each year for encephalitis. In nearly half of these cases, no specific cause can be identified. However, among cases with a known etiology, 20% to 50% are attributed to viral infections. Of these, HSV accounts for approximately 50% to 75% of identified viral cases, while the remainder are primarily associated with varicella-zoster virus (VZV), enteroviruses, and arboviruses [3].

Regarding the incidence of HSV encephalitis, it varies considerably worldwide, with reported rates ranging from 0.7 to 13.8 cases per 100,000 population across all age groups. In adults, the incidence is estimated at 0.7 to 12.6 per 100,000 persons, whereas in children it is significantly higher, ranging from 10.5 to 13.8 per 100,000. HSV encephalitis is associated with morbidity and mortality rates approaching 70%, underscoring the importance of timely diagnosis and appropriate treatment to improve patient survival [1,4].

In Latin America, the population-based incidence of pediatric viral encephalitis is not well defined in the literature, and the available data mainly derive from hospital-based studies or reports of specific outbreaks. Reported outbreak rates range from 0.1 to 2.6 per 1,000 inhabitants in affected areas, although the baseline annual incidence is likely lower and varies across countries and according to the predominant viral agent. This epidemiological uncertainty reflects the limited regional research and diagnostic surveillance in pediatric populations [5,6].

Among the most common symptoms in patients with HSV encephalitis are alterations in mental status and level of consciousness within the first 24 hours, accompanied by fever, gastrointestinal symptoms, skin rashes, confusion, hallucinations, onset of seizures, and focal neurological deficits. These deficits may include cranial nerve involvement, paraparesis, dysphasia, aphasia, or ataxia [4].

On the other hand, one of the main diagnostic tools for HSV encephalitis is polymerase chain reaction (PCR) testing of cerebrospinal fluid (CSF), which has a sensitivity of 98% and a specificity of 94% [1]. However, this test may yield false-negative results during the first four days of the disease course. For this reason, it is recommended to repeat the test after the third or fourth day to confirm the diagnosis accurately [7]. The objective of this study is to present a detailed description of a case of viral encephalitis in a young girl, from diagnosis through clinical course and treatment.

## Case presentation

A six-year-old female patient, previously healthy, fully immunized, and with no comorbidities, presented to the emergency department with an acute episode that began less than 24 hours before admission, characterized by fever, headache, vomiting, and progressive somnolence. The fever resolved spontaneously prior to hospital arrival. Within a few hours, she developed a generalized tonic-clonic seizure lasting approximately 20 minutes, followed by drowsiness and disorientation. She was transferred to Clínica Bíblica, San José, Costa Rica for neurological evaluation and further diagnostic workup. The patient also had a history of occasional episodes of bronchial hyperreactivity, managed symptomatically without fixed treatment.

On physical examination, the patient was afebrile, with a blood pressure of 127/84 mmHg, heart rate of 118 bpm, respiratory rate of 23 breaths per minute, and oxygen saturation of 97%. Reported symptoms included severe headache, vomiting, and somnolence. Physical examination findings (signs) revealed the presence of oral aphthae, mild lower lip edema likely secondary to a bite injury during the seizure, and no focal neurological deficits. Neurological examination showed somnolence without meningeal irritation or focal motor signs. The oral aphthae were considered nonspecific and not consistent with herpetic gingivostomatitis.

Table 1 shows her initial biochemical profile, which revealed mild hyponatremia, mild hyperglycemia, and mild hyperkalemia.

**Table 1:** Initial biochemical profile of the patient upon hospital admission ALT: alanine aminotransferase, also referred to as Transaminase Glutâmico-Pirúvica (TGP); AST: aspartate aminotransferase, also referred to as Transaminase Glutâmico Oxalacética or TGO; CRP: C-reactive protein.

Parameter	Result	Reference range	Interpretation
Sodium	133.6 mmol/L	136-145 mmol/L	Mildly decreased
Potassium	5.32 mmol/L	3.5-5.1 mmol/L	Mildly elevated
Magnesium	2.06 mg/dL	1.7-2.1 mg/dL	Normal
Calcium	9.64 mg/dL	8.8-10.8 mg/dL	Normal
Glucose	104 mg/dL	65-99 mg/dL	Mildly elevated
ALT (TGP)	13.6 U/L	5-32 U/L	Normal
AST (TGO)	28.3 U/L	0-35 U/L	Normal
Creatinine	0.43 mg/dL	0.51-0.95 mg/dL	Mildly decreased
CRP	0.551 mg/L	<8.0 mg/L	Normal

Given the clinical presentation and the severity of the prolonged seizure, she was hospitalized, and empirical intravenous acyclovir therapy was initiated immediately, without waiting for confirmatory laboratory or imaging results, while complementary studies, including complete blood count (CBC), chest X-ray, and extended metabolic panel, were performed. All were within normal limits.

**Figure 1:**
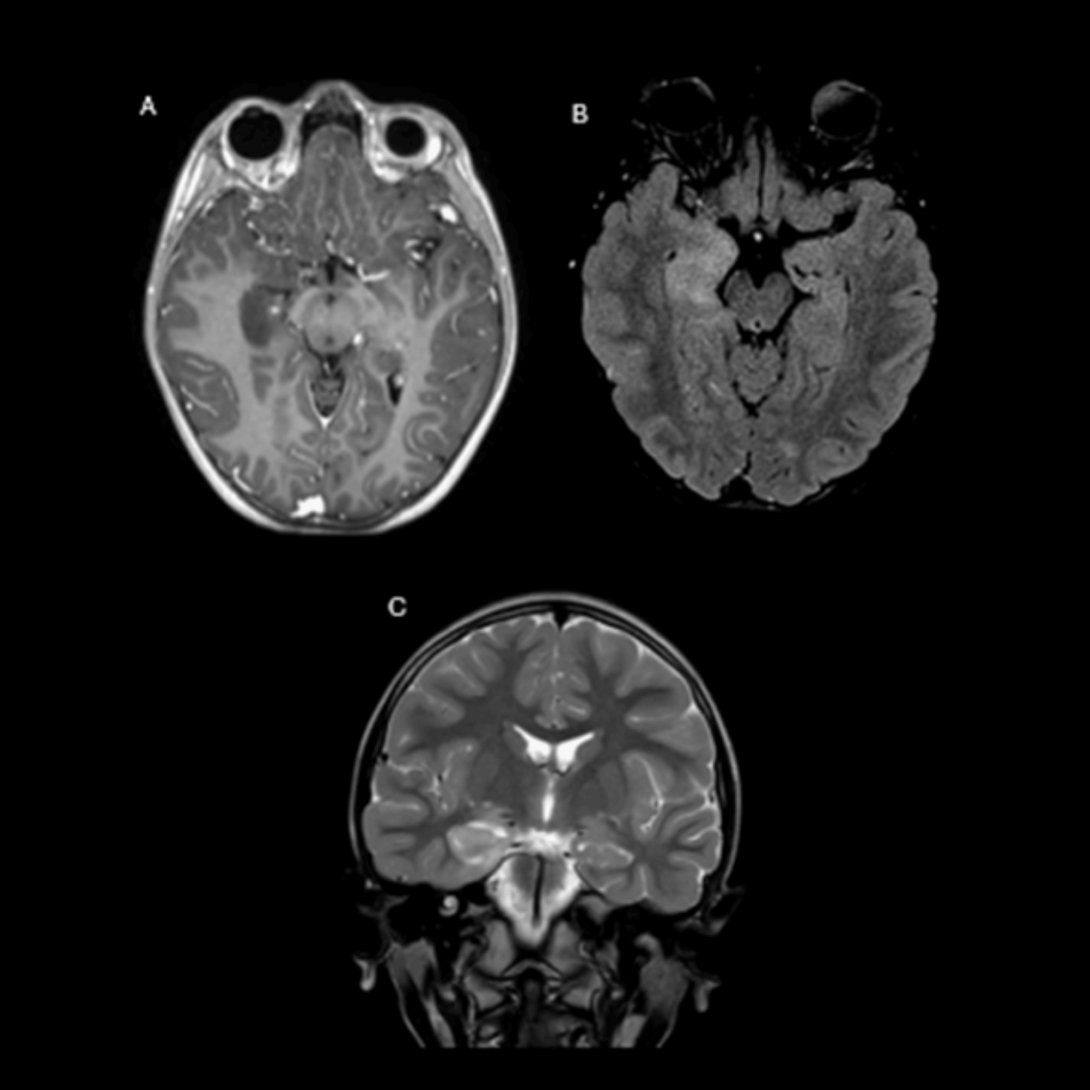
Brain magnetic resonance imaging (A) Axial T1-weighted contrast-enhanced sequence: hypointensity is observed in the right hippocampus with volume enlargement due to edema, without enhancement following paramagnetic contrast administration; (B) Axial fluid-attenuated inversion recovery (FLAIR) sequence: demonstrates diffuse hyperintensity from edema in the mesial temporal cortex, with particular involvement of the right hippocampus and parahippocampal gyrus; (C) Coronal T2 sequence: confirms unilateral involvement of the right mesial temporal structures, with no contralateral involvement.

Brain magnetic resonance imaging (MRI) performed on day one of hospitalization revealed findings characteristic of herpetic encephalitis. A unilateral involvement pattern was observed in the right mesial temporal region, affecting both the hippocampus and parahippocampal gyrus. On the axial T1-weighted sequence with contrast (Figure 1A), hypointensity was identified in the right hippocampus with volume enlargement due to edema, without enhancement following contrast administration.

Subsequently, the axial fluid-attenuated inversion recovery (FLAIR) sequence (Figure 1B) and coronal T2 sequence (Figure 1C) confirmed diffuse hyperintensity in the right mesial temporal cortex, particularly involving the hippocampus and parahippocampal gyrus, with no evidence of contralateral involvement. This anatomical distribution was highly suggestive of HSV-1 encephalitis and corresponded to a classic presentation described in the literature. 

Video electroencephalography (EEG) performed during hospitalization showed wakefulness with slow background activity interspersed with transient sharp waves and rhythmic 3-4 Hz high-voltage discharges, without spike or polyspike paroxysms. These findings indicated diffuse cortical dysfunction, consistent with an ongoing encephalitic process and concordant with the MRI abnormalities.

On day two of hospitalization, the CSF analysis revealed findings compatible with early-stage viral encephalitis. PCR testing was positive for HSV-1, confirming the diagnosis (Table 2).

**Table 2:** CSF and PCR analysis on the second day of hospitalization HSV: Herpes Simplex Virus; CSF: Cerebrospinal fluid; PCR: Polymerase chain reaction.

Parameter	Result	Reference range	Interpretation
HSV-1 PCR	Detected	Negative	Positive
CSF Glucose	60.9 mg/dL	50-80 mg/dL	Normal
CSF Protein	23.6 mg/dL	15-60 mg/dL	Normal
CSF Leukocytes	<1 cell/μL	0-5 cells/μL	Normal
CSF Erythrocytes	15 cells/μL	0	Probable mild hemorrhage

Despite virological positivity, glucose, protein, and leukocyte levels remained within normal ranges, a pattern not uncommon in the early phase of HSV-1 encephalitis. The presence of 15 erythrocytes/µL suggested mild meningeal hemorrhage, a feature occasionally observed in temporal lobe involvement. These results highlight that normal CSF biochemical parameters do not exclude HSV-1 encephalitis, particularly in early or moderate immune responses.

## Discussion

Herpetic encephalitis is characterized by a well-established anatomical predilection for the temporal lobes, particularly the mesial regions such as the hippocampus and parahippocampal gyrus [8]. This affinity reflects the neurotropism of HSV-1 and accounts for both the neurological symptoms and the typical neuroimaging patterns. In this case, MRI revealed unilateral hyperintensity in the right hippocampus, without contralateral involvement or abnormal contrast enhancement, a classic finding in the early stages. Recent studies have shown that the number and extent of FLAIR lesions may correlate with functional prognosis, with unilateral lesions, as seen in this patient, potentially indicating a more favorable clinical outcome [9].

Complementarily, EEG serves as a valuable tool to reinforce diagnostic suspicion and provide prognostic information. In herpetic encephalitis, background activity typically demonstrates focal slowing in the temporal regions, reflecting the preferential anatomical involvement of the temporal lobe by HSV-1. In severe cases, this pattern may be accompanied by diffuse disorganization and suppression episodes, which are associated with a poorer prognosis [10]. Furthermore, an anatomical correlation has been documented between focal abnormalities detected on EEG and hyperintense lesions on T2/FLAIR magnetic resonance imaging, supporting its diagnostic utility even in scenarios where neuroimaging shows only subtle changes [11].

These imaging findings correlated with the patient’s clinical presentation, which was characteristic of acute viral encephalitis, including fever, headache, altered level of consciousness, and a prolonged febrile seizure. This constellation of signs and symptoms is well described in the medical literature as representative of the encephalitis syndrome, although it does not allow for a definitive distinction between HSV-1 and other viral etiologies, particularly in the early stages. Nonetheless, the acute presentation, combined with the temporal imaging findings, strengthened the clinical suspicion of herpetic encephalitis [3].

The clinical and imaging findings were consistent with herpetic encephalitis, supporting the appropriateness of initiating empirical antiviral therapy. The treatment of choice for HSV-1 encephalitis is intravenous acyclovir, which should be started immediately upon clinical suspicion, without waiting for virological confirmation. Current evidence and clinical guidelines emphasize that early initiation of empirical acyclovir therapy is crucial to prevent severe neurological sequelae and reduce the mortality associated with this condition [12].

Several sources recommend initiating acyclovir in children at a dose of 10-20 mg/kg intravenously every eight hours, to be continued for a period of 14 to 21 days depending on clinical evolution and therapeutic response [13,14]. In the present case, in accordance with international guidelines and the recommendations of the pediatric infectious disease team, acyclovir was administered at a weight-adjusted dose of 20 mg/kg (equivalent to 500 mg) intravenously every eight hours, starting with a single loading dose on the first day. The treatment was completed over 21 days without interruptions, showing good tolerance and no adverse effects related to the antiviral. This therapeutic approach effectively controlled the infection, prevented progression of neurological damage, and led to full clinical recovery [1,2,13].

Although mortality from HSV-1 encephalitis in pediatric patients is generally low, outcomes depend largely on early diagnosis and initiation of antiviral therapy. The risk of poor prognosis is higher in neonates and infants under 12 months of age, especially when encephalitis presents as a part of disseminated infection [1,13].

While some children may be discharged from the hospital without overt clinical evidence of sequelae, HSV-1 central nervous system (CNS) infection has been associated with neurological impairments that may manifest later. For this reason, several experts have emphasized the importance of long-term neurological follow-up, ideally extending into young adulthood, to enable early detection of any alterations in cognitive, motor, or behavioral development [1,13].

Compared to other reports from Latin America, where delays in diagnostic imaging or PCR testing are common due to limited access, this case highlights the benefit of rapid clinical decision-making and interdisciplinary coordination in improving prognosis. Few pediatric HSV-1 encephalitis cases have been reported in the region, and most describe unfavorable outcomes or residual neurological deficits. Thus, this case contributes valuable evidence regarding successful management in a resource-limited Latin American context.

## Conclusions

Herpetic encephalitis remains one of the most severe causes of acute viral encephalitis, particularly in the pediatric population, where its course can be fulminant if not promptly identified and treated. This case underscores the importance of recognizing characteristic clinical and radiological patterns, such as mesial temporal lobe involvement and acute neurological presentation, to facilitate early diagnosis and intervention.

A comprehensive approach combining MRI, EEG, CSF analysis, and immediate empirical antiviral therapy allowed for successful infection control and prevention of neurological sequelae in this patient. From a clinical standpoint, this case reinforces the need for clinicians, especially in resource-limited Latin American settings, to initiate empirical acyclovir therapy promptly when HSV-1 encephalitis is suspected, even before confirmatory testing.

Furthermore, it highlights the regional necessity for multicenter research to improve diagnostic timelines, therapeutic strategies, and long-term neurological outcomes in pediatric HSV-1 encephalitis. 
